# Inflammation Score System using Preoperative Inflammatory Markers to Predict Prognosis for Hepatocellular Carcinoma after Hepatectomy: A Cohort Study

**DOI:** 10.7150/jca.45274

**Published:** 2020-06-16

**Authors:** Qinjunjie Chen, Fengwei Li, Chengqian Zhong, Yiran Zou, Zheng Li, Yuzhen Gao, Qifei Zou, Yong Xia, Kui Wang, Feng Shen

**Affiliations:** 1 Department of Hepatic Surgery IV, the Eastern Hepatobiliary Surgery Hospital, Second Military Medical University, Shanghai, China.; 2 Department of Hepatic Surgery II, the Eastern Hepatobiliary Surgery Hospital, Second Military Medical University, Shanghai, China.; 3 Longyan First Hospital, Affiliated to Fujian Medical University, Longyan, China.; 4 Department of Molecular Diagnosis, Clinical Medical College, Yangzhou University, Jiangsu, China.

**Keywords:** inflammatory marker, liver resection, hepatocellular carcinoma, prognosis

## Abstract

**Background:** This study developed a novel inflammation score system to predict survival outcomes using preoperational inflammatory markers in hepatocellular carcinoma (HCC) after surgery.

**Materials and Methods:** An inflammation score system was developed using five preoperative inflammatory markers based on the clinical data of 455 HCC patients (training cohort) receiving radical resection in the Eastern Hepatobiliary Surgery Hospital. The system was validated using a cohort from a different hospital (external validation). Kaplan-Meier curves and log-rank test were used to compare the survival of patients with different inflammation scores. A nomogram including inflammation scores for survival prediction was created to exhibit the risk factors of overall survival (OS).

**Results:** The patients in the low-score group showed better OS and recurrence-free survival (RFS) in the training and external validation cohorts than those from the high-score group. Subgroup analysis showed that compared with patients in the training cohort from the high-score group, stage I (eighth TNM stage) patients in the low-score group exhibited better prognosis results, whereas the findings for Stage II and III patients were different. Multivariate Cox analysis revealed that high inflammation score is an independent risk factor of OS and RFS. The nomogram established using the inflammation score with the C-index value of 0.661 (95% confidence interval=0.624-0.698) revealed a good three- and five-year calibration curves.

**Conclusions:** The inflammation score system based on five preoperative inflammatory markers well predicted the survival of HCC patients after surgery, especially in those at the early stage (Stage I).

## Introduction

Hepatocellular carcinoma (HCC) remains a significant health problem in the world; it is the sixth most common neoplasm and the third leading cause of cancer-related mortality worldwide 1-3. At present, the prognosis of HCC remains dismal, and the long-term prognosis after HCC resection remains unsatisfactory because of the high recurrence rate of up to 60% to 70% in patients within five years after surgery 4. Thus far, surgical resection is one of the best curative treatments for patients with early-stage HCC. However, only 10%-37% of patients are suitable for curative resection, and the prognosis results, such as overall survival (OS), recurrence-free survival (RFS), or disease-free survival, remain dissatisfactory 5, 6. Thus, effective prognostic biomarkers are required to predict the prognosis of patients with HCC before hepatectomy.

Numerous studies have demonstrated that inflammation is a decisive component of tumor progression. Several inflammatory markers, such as modified Glasgow prognostic score, platelet-to-lymphocyte ratio (PLR), and neutrophil-to-lymphocyte ratio (NLR), have been proven to be significant prognostic factors in many cancers 7-9. Evidently, high pretreatment PLR and NLR promote tumor spread, tumor cell invasion, and adhesion 10. In addition, many studies have established the prognostication value of a new cancer-related inflammatory system, that is, the combination of inflammation indices on many malignant tumors, such as non-small cell lung cancer, gastric cancer, and nasopharyngeal cancer 11-13. We developed a novel inflammation-based score system to predict survival outcomes using five preoperational inflammatory markers in HCC after surgery.

Nomogram is a novel tool or a new standard that predicts the OS of many cancer patients 14. Here, we constructed a prognostic nomogram concerning preoperative inflammation score for post-operation HCC patients.

## Material and Methods

### Patients

Data of 455 HCC patients from September 2010 to September 2012 as training cohort form the Eastern Hepatobiliary Surgery Hospital (EHBH) and 253 HCC patients from October 2010 to October 2013 as an external validation cohort form Longyan First Hospital were retrospectively reviewed. The inclusion criteria were as follows: (1) radical resection, (2) no anticancer treatment before surgery, (3) no history of other malignant diseases, (4) Child-Pugh grade A or B7 liver function, (5) no evidence of distant metastasis and major portal vein/hepatic vein invasion. Patients who received palliative tumor resection or combined other malignant diseases, had incomplete data, and failed to follow-up within 30 days from the date of surgery were excluded. Patients in the external validation cohort were assessed with the same inclusion/exclusion criteria. Informed consent was obtained from all the patients before surgery.

### Preoperative examination and hepatectomy

All patients were routinely investigated with liver and renal function tests, blood routine tests, carbohydrate antigen 19-9 (CA19-9), carcinoembryonic antigen (CEA), serum alpha-fetoprotein (AFP), chest X-ray, abdominal ultrasound, contrast-enhanced computerized tomography (CT) scan, or/and magnetic resonance imaging (MRI) of the abdomen. We also specifically collected the data of pre- and post-operative platelets, neutrophils, and lymphocyte cell count. The preoperative diagnosis of HCC was based on the criteria of the American Association for the Study of Liver Diseases 15.

The inflammatory markers studied in this research contained aspartate aminotransferase (AST)-to-alanine aminotransferase (ALT) ratio (AAR), AST-to-lymphocyte ratio index (ALRI), PLR index (PLR), NLR ratio index (NLR), AST-to-neutrophil ratio index (ANRI), and AST-to-platelet-count ratio index (APRI).

All patients underwent liver resection (LR) with the intention of complete removal of macroscopic tumors with adequate resection margins. The scheme of hepatectomy was carefully developed through a preoperative assessment. The patients were recommended for LR if they were in good general condition, with technically resectable tumors, and showed sufficient estimated volume of the future liver remnant. Histopathologic study of the surgical specimens was carried out independently by three pathologists, who came to a consensus by discussion if any controversy existed. Major hepatectomy was defined as the removal of ≥3 Couinaud liver segments 16.

### Clinicopathologic variables

Patient age at the time of operation, gender, and Child-Pugh classification were regularly measured. The tumor-related variables based on histopathological examination include tumor size, number, and presence of microvascular invasion (MVI). Tumor number was classified as solitary or multiple, and the largest diameter was measured for patients with multiple tumors.

Commonly used scoring systems contained the 8th edition of the American Joint Committee on Cancer (AJCC) 17, Child-Pugh stage 18, Barcelona Clinic Liver Cancer (BCLC) stage 3, Cancer of the Liver Italian Program (CLIP) stage 19 and Chinese University Prognostic Index (CUPI) score 20. We used the future liver remnant (FLR) volume to assess the residual effective volume of a liver after resection which was automatically calculated by the image analysis software in the computer-based CT scan. Standard liver volume (SLV) refers to the stable liver volume in each adult under physiological conditions, and its size depends on the body surface area (BSA). The SLV was calculated using the equation SLV (ml) = 706.2 × BSA (body surface area, m2) + 2.4 21. BSA was calculated using the equation BSA (m^2^) = weight (kg)^0.425^×height (cm)^0.725^×0.007184 22. And then we use the standardized future liver remnant (sFLR) = FLR/SLV to represent the percentage of the liver that will remain after hepatectomy.

### Follow-up

All the patients were followed-up regularly after discharge using the following protocol. Briefly, the assessments were conducted once every two months in the first two years after surgery and then once every three to six months with blood routine tests, prothrombin time (PT), liver function tests, levels of CA19-9, CEA, AFP, and abdominal ultrasound. A contrast-enhanced CT scan or/and MRI was performed once every six months or earlier if tumor recurrence/metastasis was suspected. The diagnosis of HCC recurrence was similar to that of the initial disease diagnosis. Recurrent lesions were managed by a multidisciplinary approach as previously reported 23. Death and recurrence were used as endpoints. OS and RFS were applied as the main prognostic parameters. OS was defined as the interval between the date of LR to the date censored, the date of the patient's death, or the last follow-up. RFS was measured from the date of LR to the date of the first HCC recurrence or last follow-up.

### Cut-off value and inflammation score

Time-dependent areas under the receiver operating characteristic (ROC) curve (AUC) of each point in different indicators were measured from 20 months to 100 months after surgery, thereby reflecting the performance in predicting OS at various time points. In this study, the five-year OS rate was used as an endpoint to stratify the values of AAR, ALRI, PLR, NLRI, ANRI, and APRI through the ROC curves. The cut-off point representing the highest Youden index (specificity + specificity-1) was selected as the optimal threshold value 24. As shown in table S1, exp(coefficient) was the weight of inflammatory markers. The method of scoring each patient was as follow, for example, when a patients' AAR value is more than 0.964, the score of AAR is 1.583 point (1.583* 1). If his or her AAR value less than or equal to 0.964, the score is zero (1.583 * 0) and so on.

### Statistical analysis

Continuous variables with normal distribution were reported as the mean and standard deviation (mean ± SD), and the median and interquartile range [median (Q_L_, Q_U_)] were used without normal distribution. The differences were compared by Student's *t* test and Mann-Whitney *U* test. Categorical variables were reported as the number of cases and percentages; the differences between the three groups were compared by the chi-square test. The Kaplan-Meier (KM) method was used to analyze OS and RFS to recurrence, and the differences were explored by the log-rank test. The Cox proportional hazard model was used to analyze the independent prognostic factors of prognosis. We used survival tree analysis to define the best cut-off value of inflammation score and then form two differentiated groups of patients (high-score group and low-score group) with different prognosis. The nomogram was built based on the results of multivariate Cox regression analyses of OS with enter method by using the rms package of R, version 3.2.0 (http://www.r-project.org/). We compared the nomogram's ability of survival prediction with other commonly used scoring systems, such as AJCC 8th TNM stage, Child-Pugh stage, BCLC stage, and so on by the time-dependent areas under the ROC curve of each point in different cohorts from 10 months to 60 months. Variables with P values < 0.05 in the univariate analysis were included in the multivariate analysis. Statistical analysis was performed using the SPSS® version 25.0 (IBM, Armonk, New York, USA). R program version 3.2.0 (http://www.r-project.org/) was used, with *P* value less than 0.05 indicating statistical significance.

## Results

### Inflammation score system

**Figure 1:**
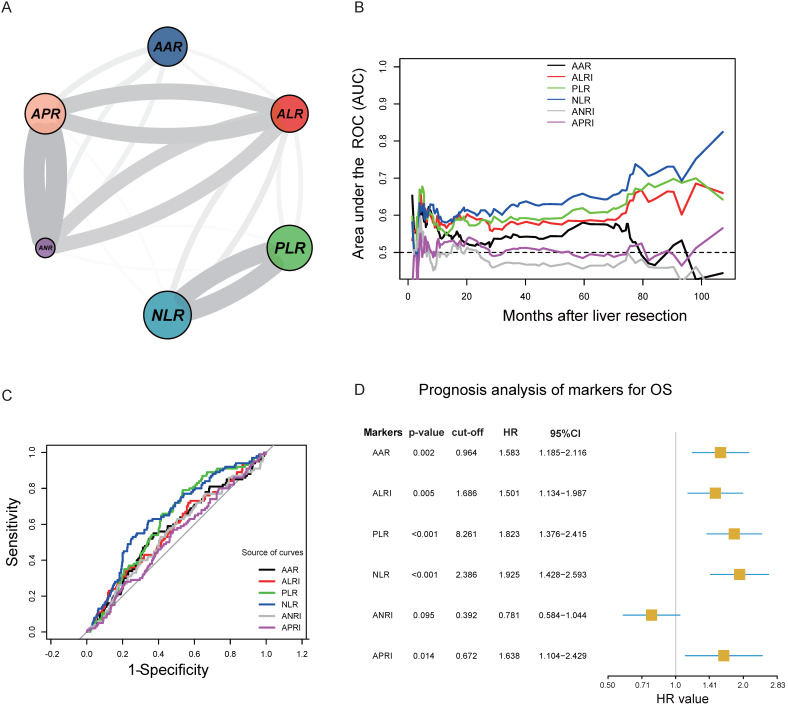
** A.** Circle correlation plot showing the relationship among the six inflammatory markers; **B.** time-dependent AUC of six indicators from 20 months to 100 months; C. cut-off values of six inflammatory markers and the prognosis analysis of markers for OS; D. ROC curves of AAR, ALRI, PLR, NLR, ANRI, and APRI.

The circle correlation plot shows the relationship among the six inflammatory markers. The gray line indicates that the coefficient value was more than 0.5 (Figure 1A). Figure 1B illustrates the time-dependent AUC ranging from 20 months to 100 months for different indicators.

The ROC curve analysis showed that the AUC of AAR, ALRI, PLR, NLR, ANRI, and APRI were 0.570, 0.571, 0.620, 0.649, 0.551, and 0.526, respectively. We identified 0.964, 1.686, 8.261, 2.386, 0.392, and 0.672 as the optimal cut-off values that provide the greatest sensitivity and specificity in the training cohort (Figure 1C). Figure 1D shows the univariable analysis results for the six inflammatory markers. We observed that five markers (AAR, ALRI, PLR, NLR, and APRI) significantly influenced the OS (P<0.05) except for ANRI. Then, we scored every patient using the five inflammatory markers according to marker' values and their coefficient values as the method illustrated in Table S1.

**Figure 2:**
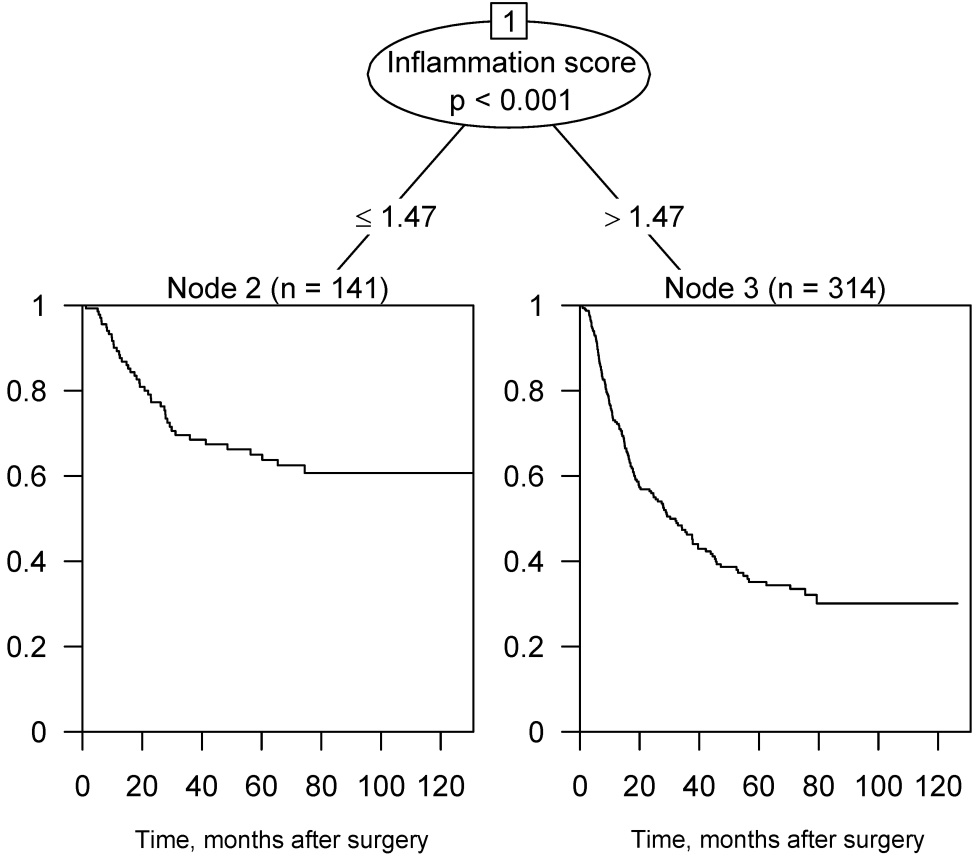
Survival tree analysis of OS in the training cohort and 1.47 points was the best cut-off value.

Figure 2 illustrates the survival tree of OS in the training cohort. According to the result, we then divided the patients into two groups using a score of 1.47 points as the criterion. In consequence, the patients with inflammation score > 1.47 were assigned to the high-score group; otherwise, they were assigned to the low-score group.

### Clinicopathological features

Table 1 shows the clinicopathologic characteristics between the high- and low-score groups with training and external validation cohorts.

### Survival analysis

**Figure 3:**
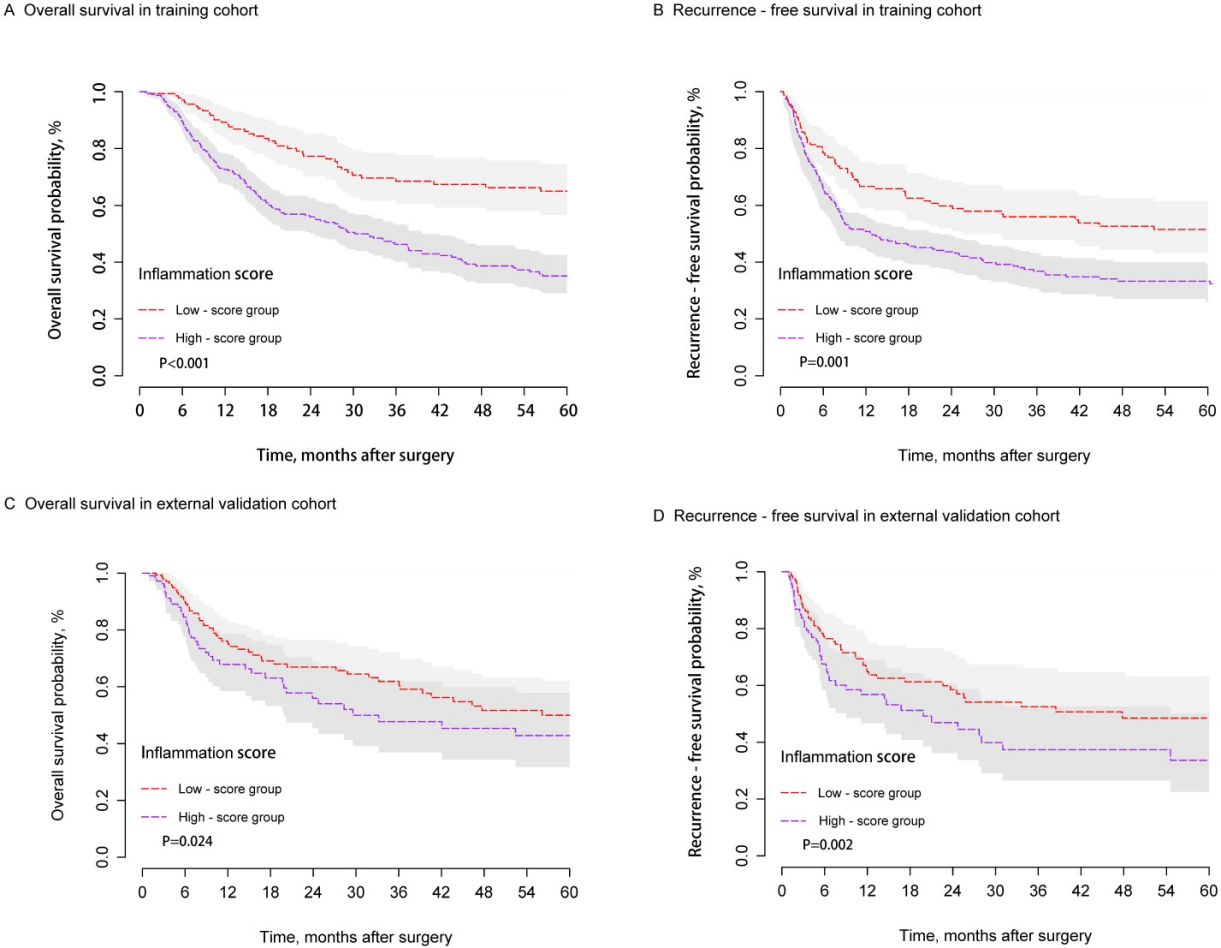
KM curves of OS and RFS for HCC patients in the training **(A and B)** and external validation cohorts** (C and D)** after hepatectomy between the high- and low-score groups.

When the study was censored (September 30, 2018), the median follow-up times were 56.2 and 31.4 months in the training and external validation cohorts, respectively. A significant difference in OS and RFS was observed between the two groups in the training cohort (Figures 3A and 3B). The postoperative one-, three-, and five-year OS rates of the low-score group (89.4%, 68.4%, and 64.9%, respectively) were significantly higher than those of the high-score group (73.8%, 47.3%, and 36.1%; P < 0.001). Similarly, the postoperative one-, three-, and five-year RFS rates of the low-score group (66.7%, 55.9%, and 51.4%; respectively) were significantly higher than those of the high-score group (53.1%, 39.6%, and 35.1%; P = 0.001). Similar results were observed in the external validation group. Patients in the high-score group presented poorer OS and RFS compared with those from the low-score group. The one-, three-, and five-year OS rates were 69.2%, 49.0%, and 44.0% for the high-score group and 77.2%, 62.8%, and 50.8% for the low-score group, respectively (P=0.024). The one-, three-, and five-year RFS rates were 49.2%, 38.1%, and 35.9% for the high-score group and 63.9%, 51.9%, and 48.3% for the low-score group (P=0.002) (Figures 3C and 3D).

The stratified analysis of the training cohort showed that Stage I patients from the high-score group exhibited poorer OS (P<0.001) rate and RFS (P=0.001) compared with those from the low-score group (Figures S1A and S1B). However, different results were observed in Stage II and III patients belonging to both groups (Figures S1C and S1D).

### Independent prognostic factors for HCC

The variables listed in the Table 1 were used for univariate Cox analysis, and the results are shown in Figures S2 and S3. Multivariate analysis showed that high inflammation score (HR=1.895, 95%CI=1.324 - 2.711, P<0.001), AFP value (HR=1.001, 95%CI=1.000 - 1.001, P=0.021), major hepatectomy (HR=1.404, 95%=1.020 - 1.934, P=0.038), and multiple tumors (HR=1.716, 95%CI=1.235 - 2.386, P=0.001) are independent risk factors of OS. High body mass index (BMI) (HR=1.007, 95%CI=1.003 - 1.011, P=0.001), high inflammation score (HR=1.522, 95%CI=1.118 - 2.072, P=0.008), AFP values (HR=1.001, 95%CI=1.000 - 1.001, P=0.005), and MVI (HR=1.504, 95%=1.093 - 2.070, P=0.012) are independent risk factors of RFS (Table 2).

### Development of a nomogram for OS prediction

**Figure 4:**
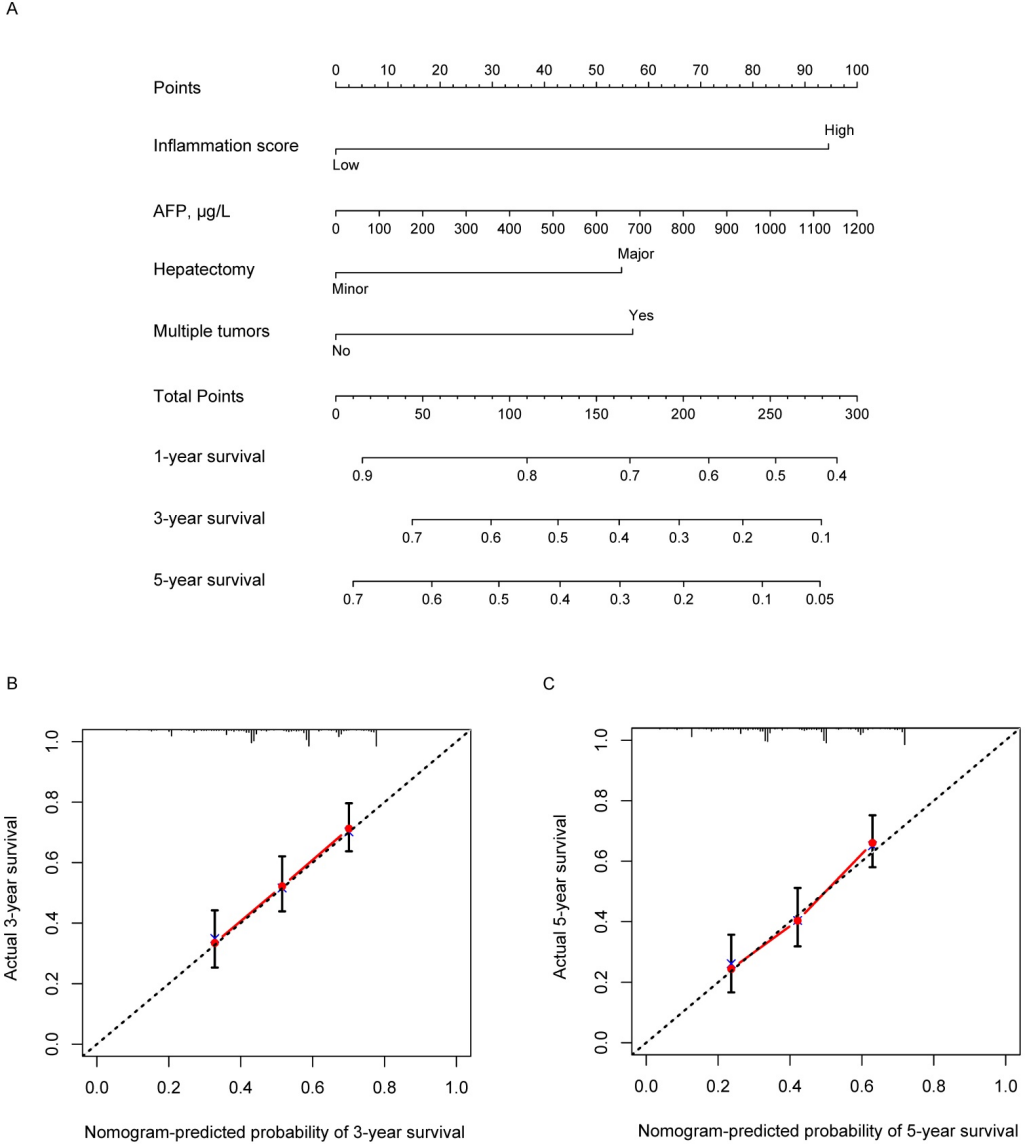
Nomogram containing inflammation score to predict the OS after hepatectomy **(A)** and the three- and five-year calibration curves **(B and C)**.

Based on the results of multivariate analysis, we conducted a further analysis by establishing a nomogram with a C-index of 0.661 (95% confidence interval (CI) = 0.624-0.698) to predict one-, three- and five-year OS rates of an individual patient. The calibration curves of the nomogram for survival probability at three and five years after radical resection presented an optimal agreement between the prediction by nomogram and actual survival (Figure 4).

### Assessment of the nomogram

**Figure 5:**
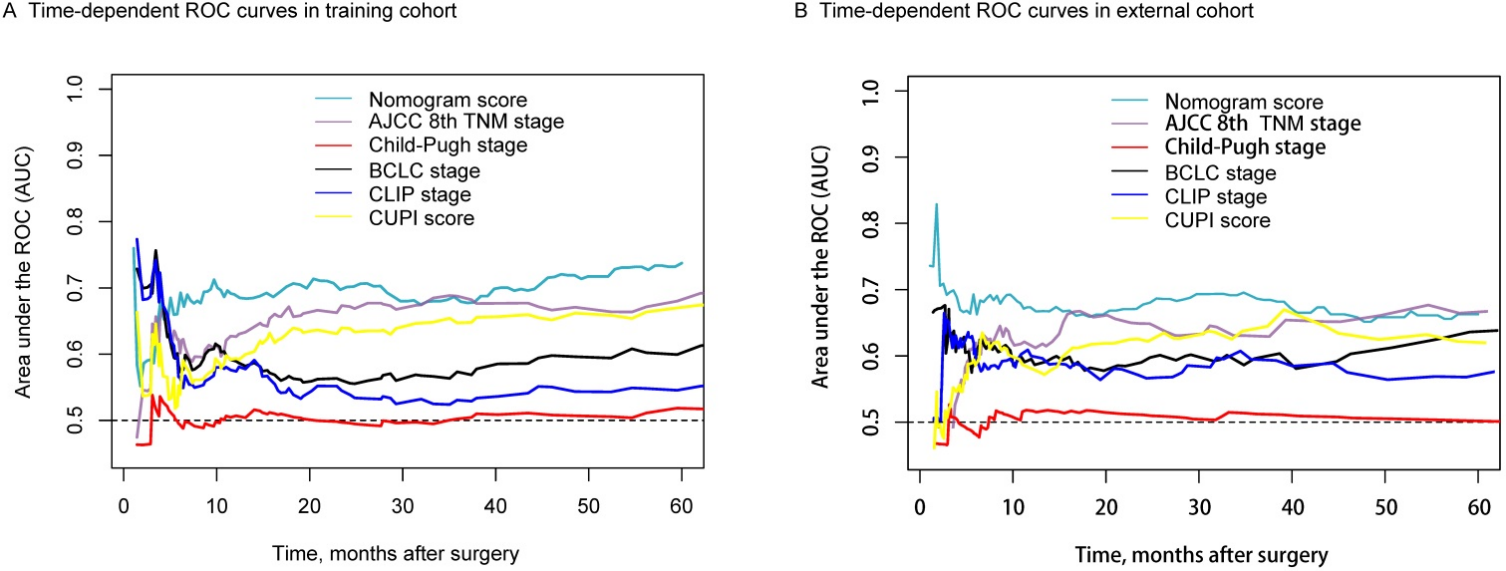
Time-dependent ROC for the nomogram model and other clinical staging systems in the training **(A)** and external validation cohorts **(B)**.

Figure 5A shows that the AUC value of the nomogram was better than the other four scoring systems except for the AJCC 8th TNM stage in the training cohort. The comparison results were list in Table S2. We found the survival predictive ability of the nomogram model was better than that of the Child-Pugh stage (P<0.0001), BCLC stage (P <0.0001), CLIP stage (P <0.0001), CUPI score (P=0.0122). However, the difference between the AJCC 8th TNM stage and the nomogram score was not significantly comparable (P=0.1092). Comparison results of different scoring systems in the external validation cohort were illustrated in Figure 5B. We found the time-dependent AUC value of the nomogram model was not worse than TNM staging, but higher than the other four scoring systems.

## Discussion

Over the last decade, many studies have demonstrated that several systemic inflammatory response markers, such as platelet count, NLR, and PLR, can be applied to predict the survival rates of patients suffering from malignant tumors 25, 26. A meta-analysis in 2016 demonstrated that an elevated NLR can be used to predict the survival of HCC patients treated with liver transplantation, hepatectomy, radiofrequency ablation, transcatheter arterial chemoembolization (TACE), or sorafenib 25. In addition, Wei Song et al. confirmed that elevated PLR after pretreatment may be predictive of poor prognosis in patients with HCC 27. A meta-analysis 10 in 2017 validated the importance of NLR and PLR for assessing the OS and RFS of HCC patients receiving different treatment types, including curative or palliative therapy 10. The inflammation score system was proposed in this study to select appropriate patients for surgery and optimally predict long-term survival among patients that underwent hepatectomy. A high inflammation score was found to be an independent predictor of poor survival after curative therapy, consistent with the conclusions of previous studies. To the best of our knowledge, this research may be the first time to combine five inflammatory markers as prognostic predictors of HCC patients receiving radical resection and the first to establish a nomogram including inflammation score to predict the OS after the operation.

We studied six inflammatory markers, including AAR, ALRI, PLR, NLR, ANRI, and APRI. These markers are all parts of the systemic inflammatory response and could reflect the influence of inflammatory-related characteristics. Thus, exploring whether different degrees of inflammation could lead to different prognostic outcomes after surgery is reasonable.

In the training cohort, as the cut-off values of AAR, ALRI, PLR, NLR, and APRI significantly influence the OS, we used them to develop an inflammation score system, which was then used to further divide the patients into high- and low-score groups to predict postoperative prognosis in HCC patients. The results of KM analyses suggested that the OS and RFS of the high-score group were significantly poorer than those of the low-score group in the training cohort. Then, we validated the results using the external validation cohorts and obtained the same results. This inflammation score system might reflect well the intensity of cancer-related inflammation because it consists of several different cellular components, including neutrophils, lymphocytes, and thrombocytes.

Although the exact mechanisms of why a high inflammation score is associated with a poor outcome remain unknown, the potential explanations may be as follows. First, AST and ALT are hepatocyte-predominant enzymes 28. Advanced liver disease is associated with mitochondrial injury, a feature that can substantially increase the release of AST. Second, the elevation of AST with the progression of liver fibrosis is caused by reduced AST clearance and mitochondrial injury with increased release of AST relative to ALT 29. Third, serum AST/ALT level is associated with remnant liver inflammatory necrosis 30, which facilitates the invasion and recurrence of HCC 31.

Cumulative evidence has also revealed that the mechanisms underlying the prognostic capability of inflammatory markers such as NLR and PLR are multi-aspect. First, several basic studies have demonstrated that neutrophilia, which causes inflammation, inhibits the cytolytic activity of immune cells, such as lymphocytes, activated T cells, and natural killer cells. Cancer-related inflammation can suppress antitumor immunity by recruiting immunosuppressive cells, such as myeloid-derived suppressor cells and regulatory T cells, thereby resulting in tumor progression 32. Kuang DM et al. observed that neutrophils are enriched predominantly in the peritumoral stroma of HCC tissues, which is positively associated with angiogenesis progression and poor survival in HCC patients 33.

Lee et al. showed that HCC patients with relatively high platelet count show a high risk of extrahepatic metastasis 34. The relatively high percentage of platelets secretes high levels of vascular endothelial growth factor and platelet-derived growth factor, which are major factors in angiogenesis, cell proliferation, and tumor metastasis 35. Nieswandt et al. have demonstrated that platelets might protect tumor cells from lysis mediated by natural killer cells to facilitate metastasis 36. Thus, increased platelet levels are considered to promote proliferation in normal liver tissues and HCC.

In addition, tumor-infiltrating lymphocytes (TILs) are important immune cells found within tumors and cause antitumor immune responses 37. High numbers of TILs correlate with favorable clinical outcomes 38, 39. In HCC patients, high levels of tumor-infiltrating CD4^+^ T lymphocytes are associated with a low recurrence rate and a reasonable prognosis 40. This finding suggests that PLR combined with the effects of neutrophils and lymphocytes may be predictive of prognosis in patients with HCC.

A novel nomogram was developed in this study to predict the survival rates of HCC patients who suffered from the operation. The results suggest that this predictive nomogram includes four integrated predictors. Tumor number and major hepatectomy are regarded as important indicators reflecting the prognosis of HCC in studies 17, 41. Besides, the model included laboratory indices, such as AFP, showing that continuously high AFP is significantly associated with poor survival after surgery 42, 43. The prognostic nomogram initially integrates the inflammation score as a basic parameter. In summary, the novel nomogram provides new insights and guidance for deciding which patients could receive maximum benefits from radical resection.

Our study featured several limitations. First, the inflammation score system enrolled patients originated from one medical center only, thereby easily leading to bias. More external validation cohorts from other medical centers should be enrolled to further validate the findings. Second, neutrophil, platelet, and lymphocyte levels are easily influenced by infections, inflammation in other tissues, and medications before HCC treatment. These factors might interfere with the inflammatory marker measurements. Third, other meaningful inflammatory markers should be further investigated to clarify and support our conclusions.

In conclusion, our results confirmed that the inflammation score system established in our hospital could be used as a novel significant predictor for prognostication in HCC patients, especially those at early TNM stages. This inflammation score system could be used to assist surgeons in identifying patients who might benefit from radical liver surgery.

## Supplementary Material

Supplementary figures and tables.

## Figures and Tables

**Table 1:** Demographics and clinicopathologic characteristics in training and external validation cohorts

	Training cohort (n=455)			Validation cohort (n=253)		
Variable	Low-score group	High-score group	*p-value*	Low-score group	High-score group	*p-value*
	n=141	n=314		n=145	n=108	
**Age**, year (mean ± SD)	52.54 ± 9.54	53.71 ± 10.84	0.270	53.41 ± 35.60	56.83 ± 43.50	0.492
**Age>65 year**, yes (%)	16 (11.3)	54 (17.2)	0.145	12 (8.3)	15 (13.9)	0.221
**Gender**, male, yes (%)	103 (73.0)	200 (63.7)	0.064	107 (73.8)	75 (69.4)	0.535
**BMI** [median (Q_L_, Q_U_)]	24.57 (22.07, 27.59)	23.00 (20.46, 26.56)	0.005	23.24 (20.24, 27.47)	23.75 (20.41, 26.95)	0.972
**HBsAg**, yes (%)	81 (57.4)	148 (47.1)	0.053	106 (73.1)	71 (65.7)	0.261
**Anti-HCV**, yes (%)	4 (2.8)	7 (2.2)	0.952	5 (3.4)	5 (4.6)	0.880
**TBIL**, mg/dL [median (Q_L_, Q_U_)]	1.00 (1.00, 1.00)	1.00 (1.00, 1.09)	0.006	0.78 (0.58, 0.92)	0.79 (0.62, 1.07)	0.270
**ALB**, g/L (mean ± SD)	43.12 ± 5.49	41.45 ± 6.27	0.007	42.72 ± 5.65	50.91 ± 56.74	0.085
**ALT**, IU/L [median (Q_L_, Q_U_)]	28.40 (18.40, 41.30)	31.05 (20.83, 56.15)	0.024	35.10 (26.80, 51.70)	30.95 (21.57, 46.88)	0.095
**AST**, IU/L [median (Q_L_, Q_U_)]	28.90 (22.00, 35.60)	32.20 (23.85, 53.92)	<0.001	27.90 (20.50, 35.00)	40.55 (28.00, 74.93)	<0.001
**GGT**, IU/L [median (Q_L_, Q_U_)]	54.00 (31.00, 92.00)	90.50 (49.00, 185.00)	<0.001	60.00 (34.00, 119.00)	111.00 (61.75, 222.75)	<0.001
**PLT**, ×10^9^/L [median (Q_L_, Q_U_)]	165.00 (136.00, 194.00)	182.00 (133.00, 237.00)	0.004	135.00 (109.00, 175.00)	126.50 (92.00, 179.00)	0.310
**Child-Pugh grade**, yes (%)	1 (0.7)	32 (10.2)	0.001	9 (6.2)	7 (6.5)	1.000
**AFP**, μg/L [median (Q_L_, Q_U_)]	35.60 (12.70, 141.80)	46.55 (15.10, 369.47)	0.024	18.20 (4.20, 310.00)	17.60 (3.40, 464.50)	0.996
**CEA**, μg/L [median (Q_L_, Q_U_)]	2.60 (1.50, 5.00)	2.80 (1.63, 7.08)	0.311	2.30 (1.50, 3.70)	2.40 (1.50, 3.20)	0.880
**CA19-9**, IU/mL [median (Q_L_, Q_U_)]	4.10 (2.40, 14.40)	4.40 (2.50, 19.88)	0.600	17.70 (10.50, 32.90)	18.00 (11.07, 30.80)	0.887
**Cirrhosis**, yes (%)	84 (59.6)	167 (53.2)	0.244	88 (60.7)	63 (58.3)	0.804
**sFLR** (mean ± SD)	0.63 ± 0.10	0.67 ± 0.11	<0.001	0.64 ± 0.12	0.64 ± 0.12	0.890
**Major hepatectomy**, yes (%)	36 (25.5)	102 (32.5)	0.167	47 (32.4)	28 (25.9)	0.328
**Surgery time**, h [median (Q_L_, Q_U_)]	2.00 (1.50, 2.50)	2.00 (1.50, 3.00)	0.029	2.50 (1.90, 3.00)	2.35 (1.90, 2.80)	0.468
**Clamp time**, min [median (Q_L_, Q_U_)]	16.00 (11.00, 22.00)	18.00 (11.25, 22.00)	0.727	15.00 (10.00, 19.00)	15.00 (10.75, 18.00)	0.652
**Blood transfusion**, yes (%)	14 (9.9)	73 (23.2)	0.001	30 (20.7)	27 (25.0)	0.510
**Tumor size^§^**, cm [median (Q_L_, Q_U_)]	4.60 (3.10, 6.00)	6.70 (4.23, 9.00)	<0.001	3.00 (2.20, 4.00)	3.00 (2.48, 3.80)	0.708
**Multiple tumors**, yes (%)	28 (19.9)	101 (32.2)	0.010	9 (6.2)	5 (4.6)	0.791
**MVI**, yes (%)	19 (13.5)	59 (18.8)	0.209	22 (15.2)	15 (13.9)	0.916

**Abbreviations:** low-score group: low-inflammation-score group; high-score group: high-inflammation-score group; BMI: body mass index; ALT: alanine aminotransferase; AST: aspartate aminotransferase; GGT: gamma-glutamyl transpeptidase; sFLR: standardized future liver remnant; MVI: microvascular invasion. § largest diameter for a solitary tumor; the diameter of the largest nodule for multiple tumors.

**Table 2:** Multivariable analysis for overall survival and recurrence-free survival

Variable	OS			RFS		
	HR	95% CI	*p value*	HR	95% CI	*p value*
BMI				1.007	1.003 - 1.011	0.001
Inflammation score, High vs. Low	1.895	1.324 - 2.711	<0.001	1.522	1.118 - 2.072	0.008
AFP, μg/L	1.001	1.000 - 1.001	0.021	1.001	1.000 - 1.001	0.005
Hepatectomy, Major vs. Minor	1.404	1.020 - 1.934	0.038			
Multiple tumors, Yes vs. No	1.716	1.235 - 2.386	0.001			
MVI, Yes vs. No				1.504	1.093 - 2.070	0.012

**Abbreviations:** BMI: body mass index; MVI: microvascular invasion.

## References

[1] Yang JD, Roberts LR (2010). Hepatocellular carcinoma: A global view. Nat Rev Gastroenterol Hepatol.

[2] Torre LA, Bray F, Siegel RL, Ferlay J, Lortet-Tieulent J, Jemal A (2015). Global cancer statistics, 2012. CA Cancer J Clin.

[3] Forner A, Reig M, Bruix J (2018). Hepatocellular carcinoma. Lancet.

[4] Zhang X, Li C, Wen T, Yan L, Li B, Yang J (2015). Appropriate treatment strategies for intrahepatic recurrence after curative resection of hepatocellular carcinoma initially within the Milan criteria: according to the recurrence pattern. Eur J Gastroenterol Hepatol.

[5] Kawaguchi Y, Otsuka Y, Kaneko H, Nagai M, Nomura Y, Yamamoto M (2016). Comparisons of financial and short-term outcomes between laparoscopic and open hepatectomy: benefits for patients and hospitals. Surg Today.

[6] Pang TC, Lam VW (2015). Surgical management of hepatocellular carcinoma. World J Hepatol.

[7] Shen J, He L, Li C, Wen T, Chen W, Lu C (2016). Prognostic nomograms for patients with resectable hepatocelluar carcinoma incorporating systemic inflammation and tumor characteristics. Oncotarget.

[8] Xue TC, Jia QA, Ge NL, Chen Y, Zhang BH, Ye SL (2015). Imbalance in systemic inflammation and immune response following transarterial chemoembolization potentially increases metastatic risk in huge hepatocellular carcinoma. Tumour Biol.

[9] Cescon M, Bertuzzo VR, Ercolani G, Ravaioli M, Odaldi F, Pinna AD (2013). Liver transplantation for hepatocellular carcinoma: role of inflammatory and immunological state on recurrence and prognosis. World J Gastroenterol.

[10] Zheng J, Cai J, Li H, Zeng K, He L, Fu H (2017). Neutrophil to Lymphocyte Ratio and Platelet to Lymphocyte Ratio as Prognostic Predictors for Hepatocellular Carcinoma Patients with Various Treatments: a Meta-Analysis and Systematic Review. Cell Physiol Biochem.

[11] Lin YH, Chang KP, Lin YS, Chang TS (2017). Pretreatment combination of platelet counts and neutrophil-lymphocyte ratio predicts survival of nasopharyngeal cancer patients receiving intensity-modulated radiotherapy. Onco Targets Ther.

[12] Liu W, Ha M, Yin N (2017). Combination of platelet count and lymphocyte to monocyte ratio is a prognostic factor in patients undergoing surgery for non-small cell lung cancer. Oncotarget.

[13] Wang J, Qu J, Li Z, Che X, Liu J, Teng Y (2017). Combination of platelet count and neutrophil-lymphocyte ratio as a prognostic marker to predict chemotherapeutic response and survival in metastatic advanced gastric cancer. Biomark Med.

[14] Wang L, Hricak H, Kattan MW, Chen HN, Scardino PT, Kuroiwa K (2006). Prediction of organ-confined prostate cancer: incremental value of MR imaging and MR spectroscopic imaging to staging nomograms. Radiology.

[15] Bruix J, Sherman M, Practice Guidelines Committee AAftSoLD (2005). Management of hepatocellular carcinoma. Hepatology.

[16] Couinaud C (1999). Liver anatomy: portal (and suprahepatic) or biliary segmentation. Dig Surg.

[17] Amin MB, Greene FL, Edge SB, Compton CC, Gershenwald JE, Brookland RK (2017). The Eighth Edition AJCC Cancer Staging Manual: Continuing to build a bridge from a population-based to a more "personalized" approach to cancer staging. CA Cancer J Clin.

[18] Durand F, Valla D (2005). Assessment of the prognosis of cirrhosis: Child-Pugh versus MELD. J Hepatol.

[19] A new prognostic system for hepatocellular carcinoma (1998). a retrospective study of 435 patients: the Cancer of the Liver Italian Program (CLIP) investigators. Hepatology.

[20] Leung TW, Tang AM, Zee B, Lau WY, Lai PB, Leung KL (2002). Construction of the Chinese University Prognostic Index for hepatocellular carcinoma and comparison with the TNM staging system, the Okuda staging system, and the Cancer of the Liver Italian Program staging system: a study based on 926 patients. Cancer.

[21] Urata K, Kawasaki S, Matsunami H, Hashikura Y, Ikegami T, Ishizone S (1995). Calculation of child and adult standard liver volume for liver transplantation. Hepatology.

[22] Du Bois D, Du Bois EF (1989). A formula to estimate the approximate surface area if height and weight be known. 1916. Nutrition.

[23] Wang K, Liu J, Yan ZL, Li J, Shi LH, Cong WM (2010). Overexpression of aspartyl-(asparaginyl)-beta-hydroxylase in hepatocellular carcinoma is associated with worse surgical outcome. Hepatology.

[24] Youden WJ (1950). Index for rating diagnostic tests. Cancer.

[25] Sun XD, Shi XJ, Chen YG, Wang CL, Ma Q, Lv GY (2016). Elevated Preoperative Neutrophil-Lymphocyte Ratio Is Associated with Poor Prognosis in Hepatocellular Carcinoma Patients Treated with Liver Transplantation: A Meta-Analysis. Gastroenterol Res Pract.

[26] Qi X, Li J, Deng H, Li H, Su C, Guo X (2016). Neutrophil-to-lymphocyte ratio for the prognostic assessment of hepatocellular carcinoma: A systematic review and meta-analysis of observational studies. Oncotarget.

[27] Song W, Wang K, Zhong FP, Fan YW, Peng L, Zou SB (2016). Clinicopathological and prognostic significance of platelet-to-lymphocyte ratio in patients with hepatocellular carcinoma. Oncotarget.

[28] Giannini EG, Testa R, Savarino V (2005). Liver enzyme alteration: a guide for clinicians. CMAJ.

[29] Okuda M, Li K, Beard MR, Showalter LA, Scholle F, Lemon SM (2002). Mitochondrial injury, oxidative stress, and antioxidant gene expression are induced by hepatitis C virus core protein. Gastroenterology.

[30] Tarao K, Rino Y, Takemiya S, Tamai S, Ohkawa S, Sugimasa Y (2000). Close association between high serum ALT and more rapid recurrence of hepatocellular carcinoma in hepatectomized patients with HCV-associated liver cirrhosis and hepatocellular carcinoma. Intervirology.

[31] Wang ZX, Jiang CP, Cao Y, Zhang G, Chen WB, Ding YT (2015). Preoperative serum liver enzyme markers for predicting early recurrence after curative resection of hepatocellular carcinoma. Hepatobiliary Pancreat Dis Int.

[32] Wang G, Lu X, Dey P, Deng P, Wu CC, Jiang S (2016). Targeting YAP-Dependent MDSC Infiltration Impairs Tumor Progression. Cancer Discov.

[33] Kuang DM, Zhao Q, Wu Y, Peng C, Wang J, Xu Z (2011). Peritumoral neutrophils link inflammatory response to disease progression by fostering angiogenesis in hepatocellular carcinoma. J Hepatol.

[34] Mlecnik B, Tosolini M, Kirilovsky A, Berger A, Bindea G, Meatchi T (2011). Histopathologic-based prognostic factors of colorectal cancers are associated with the state of the local immune reaction. J Clin Oncol.

[35] Bambace NM, Holmes CE (2011). The platelet contribution to cancer progression. J Thromb Haemost.

[36] Nieswandt B, Hafner M, Echtenacher B, Mannel DN (1999). Lysis of tumor cells by natural killer cells in mice is impeded by platelets. Cancer Res.

[37] Man YG, Stojadinovic A, Mason J, Avital I, Bilchik A, Bruecher B (2013). Tumor-infiltrating immune cells promoting tumor invasion and metastasis: existing theories. J Cancer.

[38] Fortes C, Mastroeni S, Mannooranparampil TJ, Passarelli F, Zappala A, Annessi G (2015). Tumor-infiltrating lymphocytes predict cutaneous melanoma survival. Melanoma Res.

[39] Gooden MJ, de Bock GH, Leffers N, Daemen T, Nijman HW (2011). The prognostic influence of tumour-infiltrating lymphocytes in cancer: a systematic review with meta-analysis. Br J Cancer.

[40] Wada Y, Nakashima O, Kutami R, Yamamoto O, Kojiro M (1998). Clinicopathological study on hepatocellular carcinoma with lymphocytic infiltration. Hepatology.

[41] Llovet JM, Bru C, Bruix J (1999). Prognosis of hepatocellular carcinoma: the BCLC staging classification. Semin Liver Dis.

[42] Tong MJ, Hsien C, Song JJ, Kao JH, Sun HE, Hsu L (2009). Factors associated with progression to hepatocellular carcinoma and to death from liver complications in patients with HBsAg-positive cirrhosis. Dig Dis Sci.

[43] Chan SL, Mo FK, Johnson PJ, Hui EP, Ma BB, Ho WM (2009). New utility of an old marker: serial alpha-fetoprotein measurement in predicting radiologic response and survival of patients with hepatocellular carcinoma undergoing systemic chemotherapy. J Clin Oncol.

