# Supplementary material for: Inflammation Score System using Preoperative Inflammatory Markers to Predict Prognosis for Hepatocellular Carcinoma after Hepatectomy: A Cohort Study
Source: J Cancer. 2020 Jun 16;11(17):4947–56. doi: 10.7150/jca.45274 (PMC7378936; doi:10.7150/jca.45274)

Contents of supplementary materials

Table S1. How to score the inflammatory markers

Table S2. Comparison of the nomogram score and other clinical staging systems in the training cohort

Figure S1. KM curves of OS and RFS for HCC patients with different TNM stages in the training cohort after hepatectomy.

Figure S2. Univariable analysis of OS in the training cohort.

Figure S3. Univariable analysis of RFS in the training cohort.

Table S1. How to score the inflammatory markers

Markers	Exp(coefficient)	Cut-off value	Value of marker	Score	Value of marker	Score
AAR	1.583	0.964	>0.964	1.583 * 1	≤0.964	1.583 * 0
ALRI	1.501	1.686	>1.686	1.501 * 1	≤1.686	1.501 * 0
PLR	1.823	8.261	>8.261	1.823 * 1	≤8.261	1.823 * 0
NLR	1.925	2.386	>2.386	1.925 * 1	≤2.386	1.925 * 0
APRI	1.638	0.672	>0.672	1.638 * 1	≤0.672	1.638 * 0

Abbreviation: Exp(coefficient)=hazard ratio; AAR: aspartate aminotransferase-to-alanine aminotransferase ratio; ALRI: aspartate aminotransferase-to-lymphocyte ratio index; PLR: platelet-to-lymphocyte ratio index; NLR: neutrophil-to-lymphocyte ration; APRI: aspartate aminotransferase-to-platelet count ratio index.

Table S2. Comparison of the nomogram score and other clinical staging systems in the training cohort

	C-index (95%CI)	Change (95%CI)	P-change
**Nomogram score**	0.661 (0.624-0.698)		
**AJCC 8th TNM stage**	0.612 (0.575-0.650)	-0.039 (-0.897 — 0.005)	0.1092
**Child-Pugh stage**	0.501 (0.483-0.520)	-0.150 (-0.193 — -0.120)	**<0.0001**
**BCLC stage**	0.564 (0.529-0.598)	-0.088 (-0.132 — -0.048)	**<0.0001**
**CLIP stage**	0.541 (0.504-0.578)	-0.110 (-0.162 — -0.067)	**<0.0001**
**CUPI score**	0.590 (0.550-0.631)	-0.061 (-0.109 — -0.014)	**0.0122**

Abbreviation: BCLC: Barcelona Clinic Liver Cancer; CLIP: Cancer of the Liver Italian Program score; CUPI: Chinese University Prognostic Index.

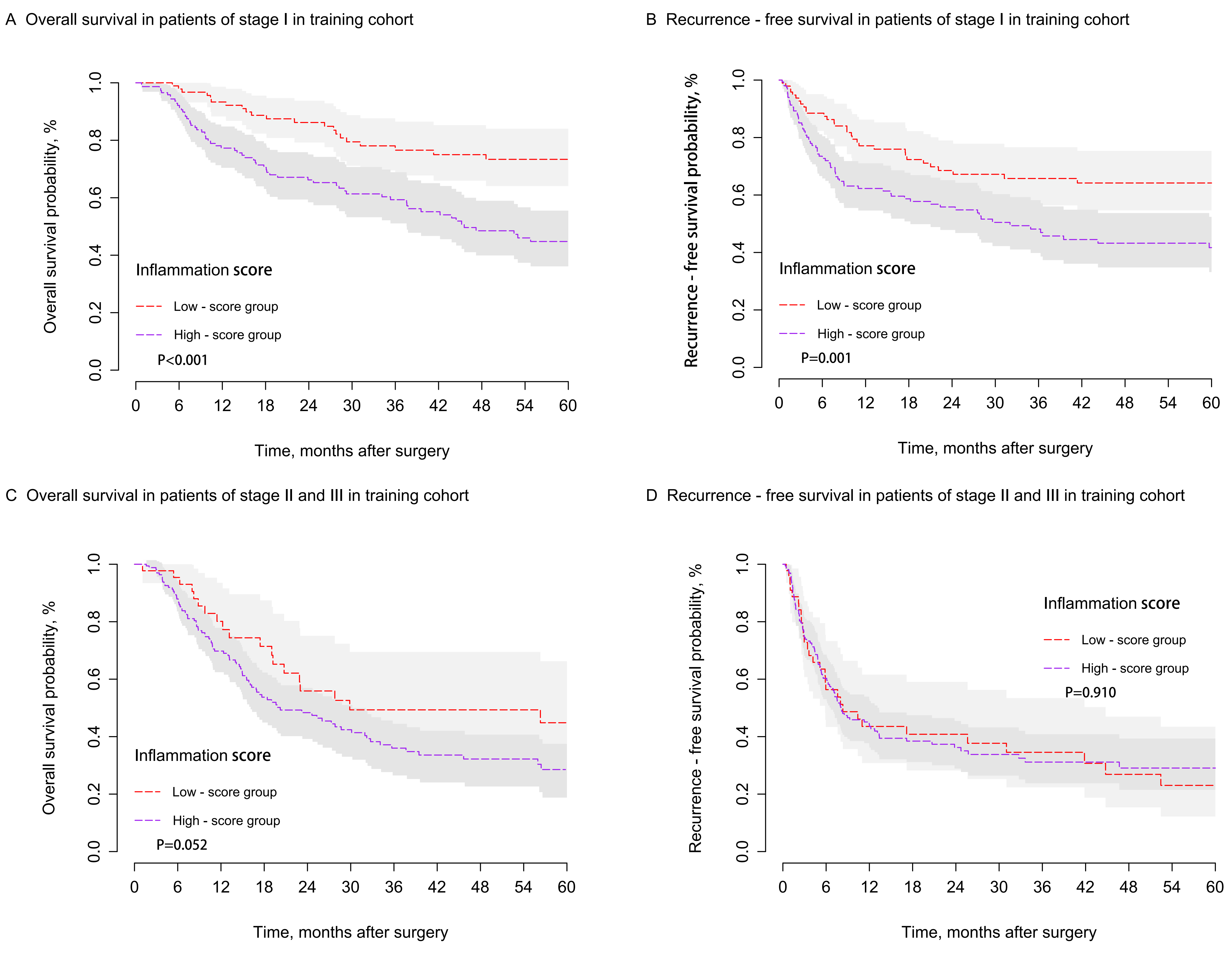
A Overall survival in patients of stage I in training cohort
Overall survival probability, %
Inflammation score
Low - score group
High - score group
P<0.001
Time, months after surgery
B Recurrence - free survival in patients of stage I in training cohort
Recurrence - free survival probability, %
Inflammation score
Low - score group
High - score group
P=0.001
Time, months after surgery
C Overall survival in patients of stage II and III in training cohort
Overall survival probability, %
Inflammation score
Low - score group
High - score group
P=0.052
Time, months after surgery
D Recurrence - free survival in patients of stage II and III in training cohort
Recurrence - free survival probability, %
Inflammation score
Low - score group
High - score group
P=0.910
Time, months after surgery

Univariate analysis of OS

Variable	95%CI	*P-value*
Age, year	1.012 (0.999-1.026)	0.074
Age, > 65y vs. ≤ 65y	1.252 (0.857-1.829)	0.244
Gender, Male vs. Female	0.805 (0.604-1.074)	0.140
BMI	1.004 (1.000-1.009)	0.051
HBsAg, Yes vs. No	0.674 (0.509-0.893)	0.006
Anti-HCV, Yes vs. No	0.897 (0.369-2.179)	0.810
TBIL, mg/dL	0.995 (0.936-1.058)	0.877
ALB, g/L	0.974 (0.952-0.997)	0.034
ALT, IU/L	1.000 (1.000-1.001)	0.255
AST, IU/L	1.000 (1.000-1.000)	0.279
GGT, IU/L	1.000 (0.999-1.000)	0.752
PLT, $\times 10^9$/L	1.001 (0.999-1.003)	0.442
Inflammation score, High vs. Low	2.353 (1.676-3.304)	<0.001
Child-Pugh grade, B vs. A	1.105 (0.641-1.903)	0.719
AFP, µg/L	1.001 (1.000-1.001)	<0.001
CEA, µg/L	1.000 (0.998-1.002)	0.840
CA19-9, IU/mL	1.000 (1.000-1.000)	0.428
Cirrhosis, Yes vs. No	0.721 (0.545-0.954)	0.021
sFLR	6.069 (1.506-24.460)	0.011
Hepatectomy, Major vs. Minor	1.623 (1.213-2.171)	0.001
Operation time, hour	1.141 (1.052-1.238)	0.002
Clamp time, minute	0.997 (0.986-1.009)	0.617
Blood transfusion, Yes vs. No	1.319 (0.940-1.850)	0.108
Tumor size, cm	1.059 (1.022-1.098)	0.002
Multiple tumors, Yes vs. No	1.520 (1.126-2.051)	0.006
MVI, Yes vs. No	1.346 (0.943-1.920)	0.100

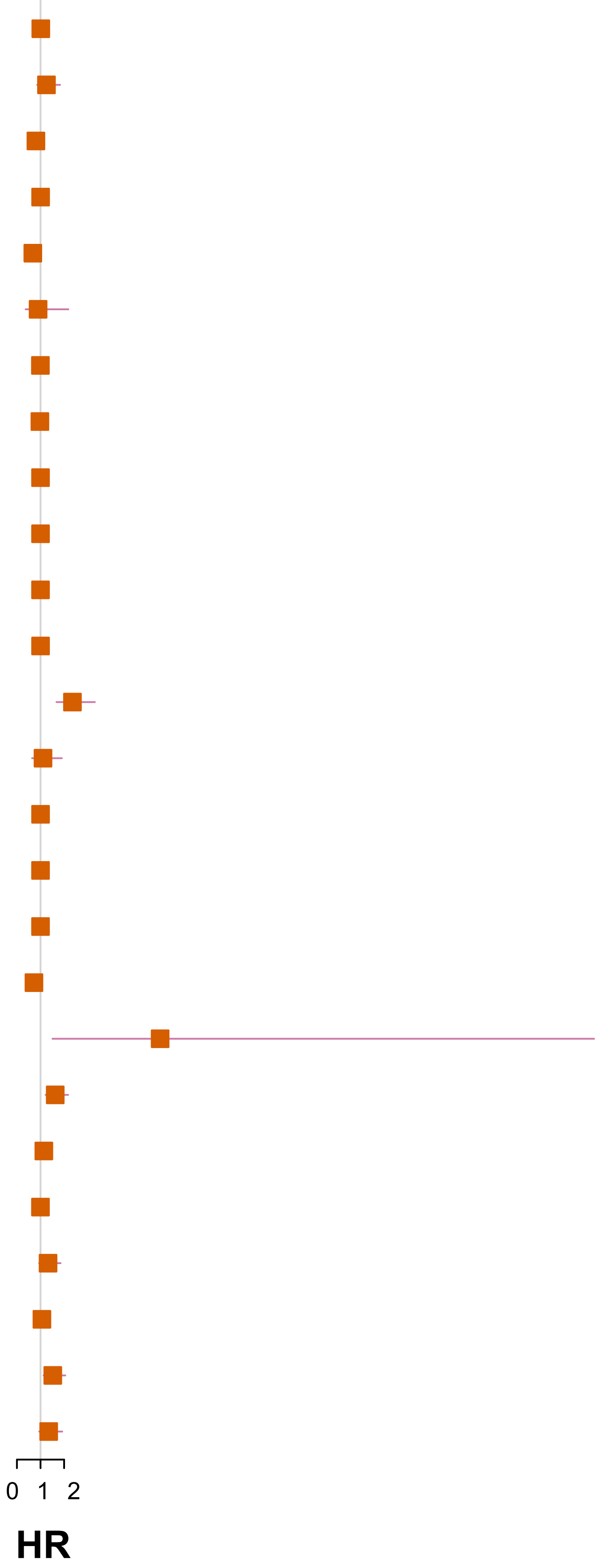

Univariate analysis of RFS

Variable	95%CI	*P-value*
Age, year	1.000 (0.989 -1.012)	0.968
Age, > 65y vs. ≤ 65y	1.245 (0.910-1.703)	0.170
Gender, Male vs. Female	0.999 (0.781-1.278)	0.995
BMI	1.004 (1.000-1.008)	0.048
HBsAg, Yes vs. No	0.814 (0.644-1.029)	0.084
Anti-HCV, Yes vs. No	1.018 (0.481-2.156)	0.963
TBIL, mg/dL	0.983 (0.935-1.034)	0.515
ALB, g/L	0.990 (0.971-1.010)	0.333
ALT, IU/L	1.000 (0.999-1.001)	0.911
AST, IU/L	1.000 (1.000-1.000)	0.340
GGT, IU/L	1.000 (0.999-1.000)	0.373
PLT, $\times 10^9$/L	1.001 (0.999-1.002)	0.525
Inflammation score, High vs. Low	1.607 (1.236-2.091)	<0.001
Child-Pugh grade, B vs. A	0.940 (0.590-1.497)	0.793
AFP, μg/L	1.001 (1.000-1.001)	<0.001
CEA, μg/L	0.999 (0.997-1.001)	0.567
CA19-9, IU/mL	1.000 (1.000-1.000)	0.346
Cirrhosis, Yes vs. No	0.814 (0.644-1.029)	0.085
sFLR	4.476 (1.479-13.548)	0.008
Hepatectomy, Major vs. Minor	1.357 (1.058-1.741)	0.016
Operation time, hour	1.037 (0.959-1.121)	0.364
Clamp time, minute	1.001 (0.993-1.010)	0.745
Blood transfusion, Yes vs. No	1.210 (0.908-1.613)	0.192
Tumor size, cm	1.052 (1.021-1.083)	0.001
Multiple tumors, Yes vs. No	1.210 (0.932-1.570)	0.152
MVI, Yes vs. No	1.659 (1.250-2.203)	<0.001

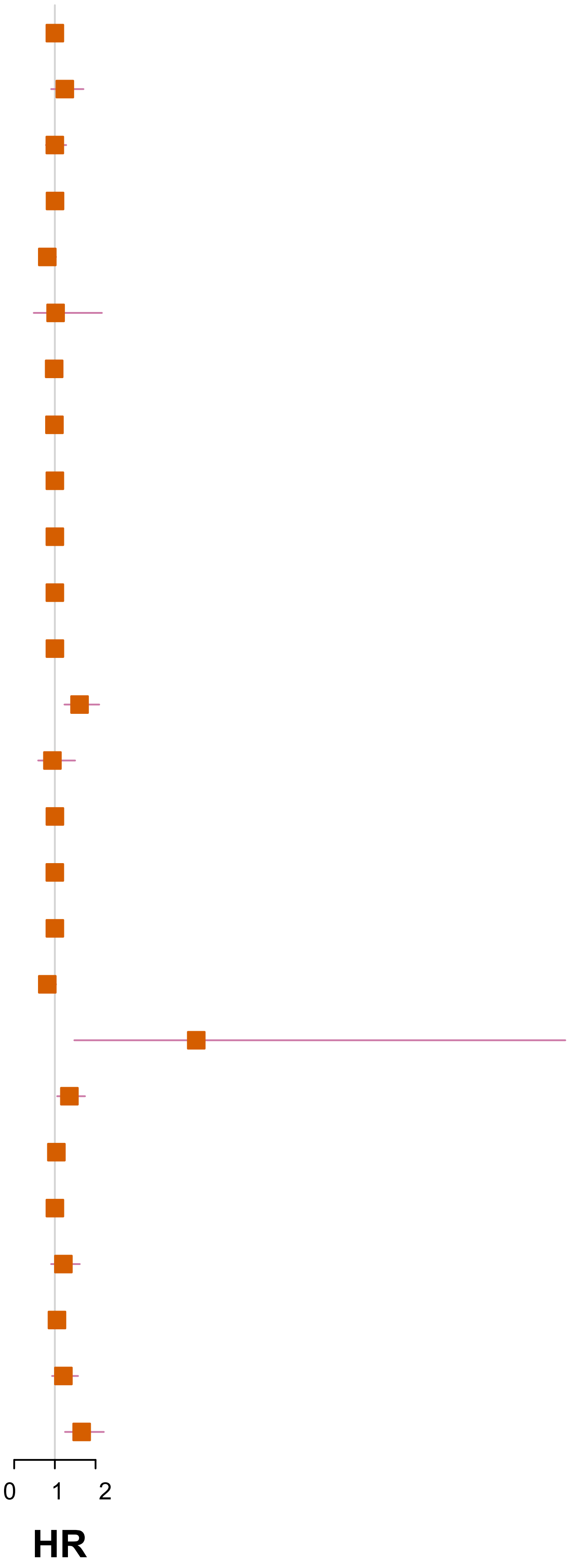

Supplement: Supplementary file 1 — Supplementary figures and tables. [file jcav11p4947s1.pdf]
